# Body weight changes in the NutriNet Brasil cohort during the covid-19 pandemic

**DOI:** 10.11606/s1518-8787.2021055003457

**Published:** 2021-03-01

**Authors:** Caroline dos Santos Costa, Eurídice Martínez Steele, Maria Alvim Leite, Fernanda Rauber, Renata Bertazzi Levy, Carlos Augusto Monteiro

**Affiliations:** I Universidade de São Paulo Núcleo de Pesquisas Epidemiológicas em Nutrição e Saúde São Paulo SP Brasil Universidade de São Paulo . Núcleo de Pesquisas Epidemiológicas em Nutrição e Saúde da USP (NUPENS). São Paulo , SP , Brasil; II Universidade de São Paulo Faculdade de Medicina Departamento de Medicina Preventiva São Paulo SP Brasil Universidade de São Paulo . Faculdade de Medicina . Departamento de Medicina Preventiva . São Paulo , SP , Brasil; III Universidade de São Paulo Faculdade de Saúde Pública Departamento de Nutrição São Paulo SP Brasil Universidade de São Paulo . Faculdade de Saúde Pública . Departamento de Nutrição . São Paulo , SP , Brasil

**Keywords:** Weight Gain, Weight Loss, Sociodemographic Variables, Nutritional Status, Coronavirus Infections

## Abstract

This study describes body weight changes among participants of the NutriNet Brasil cohort (n = 14,259) during the covid-19 pandemic. We analyzed data reported before the pandemic onset (01/26/2020 to 03/18/2020) and about six months after (09/14/2020 to 10/19/2020). Our results show that 19.7% of the participants gained ≥ 2 kg. Weight gain was directly associated with male gender, lower education, and previous presence of overweight, and inversely associated with age. In turn, 15.2% lost ≥ 2kg, being directly associated with male gender and previous presence of overweight and inversely associated with age.

## INTRODUCTION

On January 30, 2020, the World Health Organization (WHO) declared the covid-19 pandemic as a public health emergency of international concern ^a^ .

Since mid-March 2020, health authorities from Brazilian states and municipalities initiated local actions previously implemented in other countries to substantially reduce interpersonal contacts. Such actions caused most Brazilians to spend more time at home and incurred changes in many of their habits, especially diet, physical activity, and time spent watching television and using cell phones and computers, which can lead to body weight changes.

Studies showed that obesity and other obesity-related chronic non-communicable diseases increase the risk of severe covid-19, with greater need for intensive care units (ICU) treatment and death risk ^1^ . Obesity by itself is also considered a pandemic with increasing prevalence in Brazil that requires a multi-front combat. Prior to the covid-19 pandemic, obesity already inflicted substantial harm to health due to its association with diseases that lead to early death. Now, the overlap of the two pandemics raises new and alarming concerns for public health.

In this sense, understanding body weight behavior is relevant during the covid-19 pandemic. This article describes body weight changes (considering gain and loss) during the pandemic in a cohort of Brazilian adults, analyzing its association with sociodemographic variables and the presence of overweight.

## METHODS

Our data stem from the NutriNet Brasil cohort, created to prospectively investigate the association between dietary patterns and morbimortality from chronic non-communicable diseases in Brazil ^b^ . Cohort participation is voluntary, and participants consisted of individuals of at least 18 years old residing in the country. Recruitment began on January 26, 2020 through invitations issued by social networks and mass media outlet of regional and national reach. All participants register on the study’s digital platform and, every three months, answer questionnaires addressing their health status (including weight) and diet, as well as other conditions that may influence their health.

Participants who reported their weight immediately before the COVID-19 pandemic onset in Brazil (between 01/26/2020 and 03/18/2020) and about six months after (between 09/14/2020 and 10/19/2020), whose informed weight was verified less than two months before questionnaire completion, were selected for the study. When the period between the reporting of the weights was not exactly six months, weight variation was proportionally adjusted to the equivalent quantity of kilograms in the period. Individuals with outlier values for weight variation (> 200 g/day) and those who did not report height or education level were excluded, as well as pregnant women or mothers of children younger than six months (n = 372).

Prevalence of weight gain or loss equivalent to at least 2 kg within six months was calculated for the set of participants and for population strata based on potential explanatory variables of weight variation – gender, age, macro-region of residence, education level, and initial body mass index (BMI). Associations between these variables and the conditions of weight gain or loss were evaluated by calculating crude and adjusted *odds* ratios, provided by multinomial logistic regression models. A p-value <0.05 was considered statistically significant. Individuals who maintained their weight during the period or whose weight increased or decreased less than 2 kg constituted the reference category. Ordinal variables underwent linear trend tests.

## RESULTS

The Table shows the prevalence of weight gain or loss of at least 2 kg within six months for the 14,259 participants of the NutriNet Brasil cohort and for this population strata based on gender, age, macro-region of residence, education level, and initial BMI.

**Table:** Weight gain or loss of at least 2 kg within six months during the covid-19 pandemic in Brazil, according to sociodemographic variables and nutritional status at the pandemic onset. NutriNet Brasil Cohort Participants (n = 14,259).

Variables	n (%)	Weight gain ≥ 2kg in six months			Weight loss ≥ 2kg in six months		
		%	Crude OR ^b^ (95%CI)	Adjusted OR ^b^ ^,c^ (95%CI)	%	Crude OR ^b^ (95%CI)	Adjusted OR ^b^ ^,c^ (95%CI)
Gender							
Female	11,168 (78.3)	19.4	ref.	ref.	14.4	ref.	ref.
Male	3,091 (21.7)	21.1	1.18 (1.07–1.31) ^d^	1.12 (1.01–1.24) ^d^	18.1	1.38 (1.23–1.53) ^d^	1.21 (1.08–1.35) ^d^
Age group (years)							
18–24	1,345 (9.4)	25.2	ref.	ref.	12.2	ref.	ref.
25–34	3,360 (3.6)	22.9	0.93 (0.80–1.08)	0.85 (0.73–0.99)	15.6	1.30 (1.07–1.58)	1.08 (0.89–1.32)
35–44	3,928 (27.6)	20.7	0.82 (0.70–0.95)	0.68 (0.58–0.79)	16.0	1.30 (1.07–1.57)	0.93 (0.77–1.13)
45–54	2,998 (21.0)	16.9	0.62 (0.53–0.73)	0.48 (0.41–0.57)	15.7	1.20 (0.98–1.45)	0.79 (0.65–0.97)
55–64	2,140 (15.0)	14.3	0.50 (0.42–0.60)	0.38 (0.31–0.45)	14.6	1.06 (0.86–1.30)	0.69 (0.55–0.85)
≥ 65	488 (3.4)	16.0	0.56 (0.42–0.73) ^e^	0.42 (0.31–0.55) ^e^	12.5	0.90 (0.65–1.23)	0.57 (0.41–0.80) ^e^
Macro-region of residence							
North	473 (3.3)	20.9	ref.	ref.	16.7	ref.	ref.
Northeast	1,421 (10.0)	20.5	0.95 (0.73–1.24)	1.00 (0.76–1.30)	15.4	0.90 (0.68–1.20)	0.95 (0.71–1.28)
Midwest	1,136 (8.0)	20.3	0.95 (0.72–1.25)	1.02 (0.77–1.34)	16.0	0.94 (0.70–1.26)	1.01 (0.75–1.37)
Southeast	8,843 (62.0)	19.7	0.91 (0.72–1.15)	1.02 (0.81–1.30)	15.4	0.89 (0.69–1.15)	0.98 (0.75–1.27)
South	2,386 (16.7)	18.8	0.83 (0.64–1.06)	0.93 (0.72–1.20)	13.4	0.74 (0.56–0.98)	0.85 (0.64–1.13)
Education level (years)							
≥ 12	12,198 (85.6)	19.1	ref.	ref.	15.4	ref.	ref.
≤ 11	2,061 (14.5)	23.3	1.28 (1.14–1.44) ^d^	1.30 (1.15–1.46) ^d^	14.1	0.96 (0.83–1.10)	0.88 (0.76–1.01)
Overweight							
No	7,196 (50.5)	16.8	ref.	ref.	9.1	ref.	ref.
Yes	7,063 (49.5)	22.7	1.80 (1.65–1.96) ^d^	1.99 (1.82–2.18) ^d^	21.4	3.12 (2.82–3.45) ^d^	3.26 (2.94–3.62) ^d^
Total	14,259	19.7	_	_	15.2	_	_

^a^ Weight loss or gain was calculated based on participants interviewed between January 26 and March 18, 2020 and subsequently between September 14 and October 19, 2020, who reported recent weight check (maximum of two months before the interview). When the period between the interviews was not exactly six months, weight variation was proportionally adjusted to the equivalent quantity of kilograms in the period.

^b^ Reference category: individuals who maintained their weight during the period or whose weight increased or decreased less than 2 kg.

^c^ Adjustment for other covariates.

^d^ p < 0.05.

^e^ p linear trend < 0.05.

Weight gain was more prevalent than weight loss (19.7% and 15.2%, respectively) among cohort participants and all strata, except for the 55-64 age group, which presented similar prevalence for weight gain (14.3%) and loss (14.6%).

Weight gain was inversely associated with age and directly associated with male gender, lower education level (11 years or less), and previous presence of overweight (BMI ≥ 25 kg/m ^2^ ), but we found no significant association with the region of residence.

Weight loss was inversely associated with age and directly associated with male gender and presence of overweight, without significant association with education level or region of residence.

## DISCUSSION

Answers provided by over 14,000 participants of the NutriNet Brasil cohort immediately before and during the covid-19 pandemic in Brazil indicate that the prevalence of at least 2 kg weight gain within six months was 19.7%, and for weight loss it was 15.2%. Younger ages, male gender, and previous presence of overweight were risk factors for both weight gain and loss. Lower education was a risk factor for weight gain, but we verified no association between education and weight loss.

Studies conducted in Poland, France, and Spain to assess body weight changes during social isolation due to the pandemic found a higher proportion of individuals who gained weight compared to those who lost ^2^ . Weight gain frequency during the pandemic ranged from 25.8% in Spain to 35% in France, whereas weight loss frequency ranged from 18.6% in Poland to 23% in France ^2^ . We found no studies on this topic that were conducted in Brazil and included individuals from all macro-regions within the country.

Studies conducted in France and Spain verified association patterns between weight variation and sociodemographic characteristics and BMI similar to those found in the NutriNet Brasil cohort ^3 , 4^ .

Part of the association patterns found in our study (as well as in those conducted in France and Spain) may be explained by the distinct routine changes stemming from the pandemic among population strata. Individuals with more education years, for example, may have developed more favorable eating habits since they have more time to prepare meals or even more knowledge about the importance of nutrition in defense against coronavirus infection. Conversely, less educated people may have had limited access to fresh food and/or have been more affected by unhealthy foods advertising in the period ^5^ .

Body weight change decreased with age, which could be explained by the smaller change in older people’s habits, already used to staying at home longer. As for association with overweight, some participants presenting this condition may have been more concerned with health and sought to develop healthier behaviors, thus losing weight. On the other hand, participants with overweight may have been more affected by the stress imposed by the pandemic, thus gaining more weight.

Weight and height were not directly measured, but rather reported by the participants, which poses a limitation for this study. Moreover, the non-probabilistic sampling method adopted, common in cohort studies, determined a sociodemographic profile different from that expected for the adult Brazilian population, comprising a greater representation of women, individuals with high education level, and residents in the South and Southeast regions.

As for strengths, we may stress that this is the first study to assess body weight changes among Brazilians from all macro-regions of the country during the covid-19 pandemic, conducted with a large sample, and employing a before-and-after design – which allowed the same participants to answer questionnaires before and during the pandemic.

Our results indicate that weight gain was more prevalent than weight loss during the covid-19 pandemic in Brazil. Younger, male and individuals with overweight showed a greater risk of weight gain or loss during social isolation. In addition, less educated people presented a greater risk of weight gain.

## Footnotes

a World Health Organization. Coronavirus disease (COVID-19) pandemic [Internet]. Geneva: World Health Organization; 2020 [cited 2020 Dec 15]. Available from: https://www.who.int/emergencies/diseases/novel-coronavirus-2019/situation-reports

b Núcleo de Pesquisas Epidemiológicas em Nutrição e Saúde. NutriNet Brasil. São Paulo: Faculdade de Saúde Pública, Universidade de São Paulo; c2018-2020 [cited 2020 Dec 15]. Available from: https://nutrinetbrasil.fsp.usp.br
