# Supplementary material for: Body weight changes in the NutriNet Brasil cohort during the covid-19 pandemic
Source: Rev Saude Publica. 2021 Mar 1;55:01. doi: 10.11606/s1518-8787.2021055003457 (PMC9586435; doi:10.11606/s1518-8787.2021055003457)
Supplement: Translation

# Translation: Mudanças no peso corporal na coorte NutriNet Brasil durante a pandemia de covid-19

**Authors:** Caroline dos Santos Costa, Eurídice Martínez Steele, Maria Alvim Leite, Fernanda Rauber, Renata Bertazzi Levy, Carlos Augusto Monteiro

**Affiliations:** I Brasil Universidade de São Paulo. Núcleo de Pesquisas Epidemiológicas em Nutrição e Saúde da USP (NUPENS). São Paulo, SP, Brasil; II Brasil Universidade de São Paulo. Faculdade de Medicina. Departamento de Medicina Preventiva. São Paulo, SP, Brasil; III Brasil Universidade de São Paulo. Faculdade de Saúde Pública. Departamento de Nutrição. São Paulo, SP, Brasil

**Keywords:** Ganho de Peso, Perda de Peso, Variáveis Sociodemográficas, Estado Nutricional, Infecções por Coronavírus

## Abstract

Este estudo descreve modificações no peso corporal de participantes da coorte NutriNet Brasil (n = 14.259) ocorridas durante a pandemia de covid-19. Foram analisados dados informados em período anterior ao início da pandemia (26/01/2020 a 18/03/2020) e cerca de 6 meses após (14/09/2020 a 19/10/2020). O ganho de peso ≥ 2 kg alcançou 19,7% dos participantes, mostrando-se diretamente associado ao sexo masculino, à menor escolaridade e à presença prévia de excesso de peso, sendo inversamente associado à idade. A perda de peso ≥ 2kg alcançou 15,2% dos participantes, mostrando-se diretamente associada ao sexo masculino e à presença prévia de excesso de peso, sendo inversamente associada à idade.

## INTRODUÇÃO

No dia 30 de janeiro de 2020, a Organização Mundial da Saúde (OMS) declarou que a pandemia de covid-19 seria uma emergência de saúde pública de importância global^a^.

Desde meados de março de 2020, autoridades de saúde dos estados e municípios brasileiros deram início a ações locais, já implementadas em outros países, buscando a diminuição substancial de contatos interpessoais. Grande parte dos brasileiros passou a ficar mais tempo em casa e teve muitos dos seus hábitos modificados, com destaque para mudanças na alimentação, na prática de atividade física e no tempo de uso de televisão, celular e computador, o que pode levar a alterações no peso corporal.

Já há evidências de que a obesidade e outras doenças crônicas não transmissíveis relacionadas à obesidade aumentam o risco de se desenvolver formas mais severas da covid-19, com maior necessidade de tratamento em unidades de terapia intensiva e chance de morte^1^. A obesidade, também considerada por si só uma pandemia, tem prevalências crescentes no Brasil e exige um enfrentamento por múltiplas frentes. Em um cenário prévio à pandemia de covid-19, seus prejuízos à saúde, dada a associação com doenças que levam à morte precoce, são notáveis. No atual contexto, a sobreposição das duas pandemias gera novas e alarmantes preocupações na área da saúde pública.

Nesse sentido, torna-se relevante conhecer o comportamento do peso corporal durante a pandemia de covid-19. Este artigo descreve modificações no peso corporal (considerando ganho e perda) durante a pandemia entre participantes de uma coorte de brasileiros adultos, analisando sua associação com variáveis sociodemográficas e com a presença prévia de excesso de peso.

## MÉTODOS

Os dados deste estudo provêm da coorte NutriNet Brasil, criada para investigar prospectivamente a relação entre padrões de alimentação e morbimortalidade por doenças crônicas no Brasil^b^. A participação na coorte é voluntária e o recrutamento de participantes – pessoas residentes no país com pelo menos 18 anos de idade – foi iniciado em 26 de janeiro de 2020 por meio de convites divulgados em redes sociais e veículos de comunicação de massa com alcance regional e nacional. Na plataforma digital do estudo, os participantes fazem seu cadastro e, a cada três meses, respondem a questionários sobre seu estado de saúde (incluindo peso) e sua alimentação, além de outras condições que podem influenciar a saúde.

Para este estudo, foram selecionados participantes que informaram seu peso imediatamente antes do início da pandemia de covid-19 no Brasil (entre 26/01/2020 e 18/03/2020) e após cerca de seis meses (entre 14/09/2020 e 19/10/2020) e, dentre esses, aqueles que relataram que o peso informado foi averiguado há menos de dois meses da data do preenchimento do questionário. Quando o período entre o relato dos dois pesos não era de exatamente seis meses, a variação do peso foi ajustada proporcionalmente para corresponder à quantidade em quilograma equivalente ao período. Foram excluídos indivíduos com valores aberrantes para a variação do peso (> 200 g/dia) e aqueles que não informaram altura ou escolaridade, bem como mulheres grávidas ou que tiveram filho há menos de seis meses (n = 372).

Foram calculadas prevalências de ganho ou perda de peso equivalentes a pelo menos 2 kg em seis meses para o conjunto dos participantes e para estratos dessa população formados com base em potenciais variáveis explanatórias da variação do peso: sexo, faixa etária, macrorregião de residência, escolaridade e índice de massa corporal (IMC) no início do acompanhamento. Associações entre essas variáveis e as condições de ganho ou perda de peso de pelo menos 2 kg em seis meses foram avaliadas com o cálculo de *odds ratio* brutas e ajustadas fornecidas por modelos de regressão logística multinomial, adotando-se valor de p < 0,05 para identificar associações significantes. Indivíduos que mantiveram seu peso no período ou que apresentaram variações para mais ou para menos inferiores a 2 kg constituíram a categoria de referência. Testes de tendência linear foram realizados para variáveis ordinais.

## RESULTADOS

A Tabela apresenta a prevalência de ganho ou perda de peso, sempre equivalente a pelo menos 2 kg em seis meses, para o conjunto dos 14.259 participantes da coorte NutriNet Brasil incluídos no presente estudo e para estratos dessa população formados com base nas variáveis sexo, faixa etária, macrorregião de residência, escolaridade e IMC no início do acompanhamento.

**Tabela:** Ganho ou perda de peso equivalente a pelo menos 2 kg em seis meses ^a^ durante a vigência da pandemia de covid-19 no Brasil segundo variáveis sociodemográficas e estado nutricional no início do período. Participantes da Coorte NutriNet Brasil (n = 14.259).

Variáveis	n (%)	Ganho de peso ≥ 2kg em seis meses			Perda de peso ≥ 2kg em seis meses		
		%	OR bruta ^b^ (IC95%)	OR ajustada ^b^ ^,c^(IC95%)	%	OR bruta ^b^ (IC95%)	OR ajustada ^b^ ^,c^(IC95%)
Sexo							
Feminino	11.168 (78,3)	19,4	ref.	ref.	14,4	ref.	ref.
Masculino	3.091 (21,7)	21,1	1,18 (1,07–1,31)^d^	1,12 (1,01–1,24)^d^	18,1	1,38 (1,23–1,53)^d^	1,21 (1,08–1,35)^d^
Faixa etária (anos)							
18–24	1.345 (9,4)	25,2	ref.	ref.	12,2	ref.	ref.
25–34	3.360 (23,6)	22,9	0,93 (0,80–1,08)	0,85 (0,73–0,99)	15,6	1,30 (1,07–1,58)	1,08 (0,89–1,32)
35–44	3.928 (27,6)	20,7	0,82 (0,70–0,95)	0,68 (0,58–0,79)	16,0	1,30 (1,07–1,57)	0,93 (0,77–1,13)
45–54	2.998 (21,0)	16,9	0,62 (0,53–0,73)	0,48 (0,41–0,57)	15,7	1,20 (0,98–1,45)	0,79 (0,65–0,97)
55–64	2.140 (15,0)	14,3	0,50 (0,42–0,60)	0,38 (0,31–0,45)	14,6	1,06 (0,86–1,30)	0,69 (0,55–0,85)
≥ 65	488 (3,4)	16,0	0,56 (0,42–0,73)^e^	0,42 (0,31–0,55)^e^	12,5	0,90 (0,65–1,23)	0,57 (0,41–0,80)^e^
Macrorregião de residência							
Norte	473 (3,3)	20,9	ref.	ref.	16,7	ref.	ref.
Nordeste	1.421 (10,0)	20,5	0,95 (0,73–1,24)	1,00 (0,76–1,30)	15,4	0,90 (0,68–1,20)	0,95 (0,71–1,28)
Centro-Oeste	1.136 (8,0)	20,3	0,95 (0,72–1,25)	1,02 (0,77–1,34)	16,0	0,94 (0,70–1,26)	1,01 (0,75–1,37)
Sudeste	8.843 (62,0)	19,7	0,91 (0,72–1,15)	1,02 (0,81–1,30)	15,4	0,89 (0,69–1,15)	0,98 (0,75–1,27)
Sul	2.386 (16,7)	18,8	0,83 (0,64–1,06)	0,93 (0,72–1,20)	13,4	0,74 (0,56–0,98)	0,85 (0,64–1,13)
Escolaridade (anos)							
≥ 12	12.198 (85,6)	19,1	ref.	ref.	15,4	ref.	ref.
≤ 11	2.061 (14,5)	23,3	1,28 (1,14–1,44)^d^	1,30 (1,15–1,46)^d^	14,1	0,96 (0,83–1,10)	0,88 (0,76–1,01)
Excesso de peso							
Não	7.196 (50,5)	16,8	ref.	ref.	9,1	ref.	ref.
Sim	7.063 (49,5)	22,7	1,80 (1,65–1,96)^d^	1,99 (1,82–2,18)^d^	21,4	3,12 (2,82–3,45)^d^	3,26 (2,94–3,62)^d^
Total	14.259	19,7	_	_	15,2	_	_

^a^ A perda ou ganho de peso foram calculados levando em conta participantes entrevistados entre 26 de janeiro e 18 de março de 2020 e, posteriormente, entre 14 de setembro e 19 de outubro de 2020 e que informaram verificação recente do peso (no máximo até dois meses antes da entrevista). Quando o período entre as duas entrevistas não era de exatamente seis meses, a variação do peso foi ajustada proporcionalmente para corresponder à quantidade em quilogramas equivalente ao período.

^b^ Categoria de referência: indivíduos que mantiveram seu peso no período ou que apresentaram variações para mais ou para menos inferiores a 2 kg.

^c^Ajuste para as demais covariáveis.

^d^p < 0,05.

^e^p tendência linear < 0,05.

A prevalência de ganho de peso excedeu à de perda para o conjunto dos participantes (19,7% e 15,2%, respectivamente) e para todos os estratos, com exceção da faixa etária entre 55 e 64 anos, em que as prevalências de ganho ou perda de peso foram semelhantes (14,3% e 14,6%, respectivamente).

A condição de ganho de peso mostrou-se inversamente associada à idade do participante e diretamente associada ao sexo masculino, à menor escolaridade (11 anos ou menos de estudo) e à presença prévia de excesso de peso (IMC ≥ 25 kg/m^2^), não havendo associação significativa com região de residência.

A condição de perda de peso mostrou-se inversamente associada à idade e diretamente associada ao sexo masculino e à presença prévia de excesso de peso, não havendo associações significativas com a escolaridade ou com a região de residência.

## DISCUSSÃO

Com base em questionários respondidos por mais de 14 mil participantes da coorte NutriNet Brasil imediatamente antes e durante a pandemia de covid-19 no país, as prevalências de ganho e perda de peso equivalente a pelo menos 2 kg em seis meses foram estimadas em 19,7% e 15,2%, respectivamente. Idades mais jovens, sexo masculino e presença prévia de excesso de peso foram fatores de risco tanto para o ganho quanto para a perda de peso. A escolaridade mais baixa foi fator de risco para o ganho de peso, não havendo associação entre escolaridade e perda de peso.

Estudos realizados na Polônia, França e Espanha avaliaram modificações no peso corporal durante as medidas de isolamento social adotadas devido à pandemia, mostrando, também, proporções mais altas de indivíduos que ganharam peso em comparação aos que perderam^2^. A frequência de algum ganho de peso durante a pandemia variou de 25,8% na Espanha a 35% na França, enquanto a frequência de alguma perda de peso variou de 18,6% na Polônia a 23% na França^2^. Não foram encontrados estudos sobre o tema no Brasil que incluíssem indivíduos de todas as macrorregiões do país.

Os estudos realizados na França e Espanha encontraram padrões de associação da variação de peso com características sociodemográficas e IMC semelhantes ao que encontramos na coorte NutriNet Brasil^3 , 4^.

Diferenças entre estratos da população quanto a mudanças na rotina decorrentes da pandemia podem explicar parte dos padrões de associação encontrados em nosso estudo e, também, naqueles realizados na França e Espanha. Por exemplo, indivíduos mais escolarizados podem ter desenvolvido comportamentos alimentares mais favoráveis durante a pandemia por disporem de maior tempo para preparar refeições ou mesmo por terem mais conhecimento sobre a importância da nutrição na defesa contra a covid-19. Por outro lado, pessoas com menor escolaridade podem ter tido menor acesso a alimentos frescos e/ou ter sido mais afetados pela publicidade de alimentos não saudáveis no período^5^.

A diminuição na variação do peso corporal com a idade poderia ser consequência da menor variação nas formas de viver das pessoas mais velhas, já acostumadas a permanecer mais tempo em casa. Quanto à associação com excesso de peso, é possível que uma parcela dos participantes com essa condição tenha tido uma maior preocupação com a saúde e procurado desenvolver comportamentos mais saudáveis, com consequente perda de peso; por outro lado, outros participantes com excesso de peso podem ter sido mais afetados pelo estresse imposto pela pandemia, apresentando maior ganho de peso.

Entre as limitações deste estudo, destacamos que o peso e a altura não foram medidos diretamente, mas declarados pelos participantes. Além disso, a natureza não probabilística na seleção dos participantes, comum em estudos de coorte, determinou um perfil sociodemográfico distinto daquele esperado para a população brasileira adulta, com maior representação de mulheres e indivíduos com alta escolaridade e residentes nas regiões Sul e Sudeste.

Consideramos pontos fortes do estudo o fato de ser o primeiro a avaliar mudanças no peso corporal de brasileiros de todas as macrorregiões do país durante a pandemia de covid-19, o grande número de pessoas estudadas e o delineamento antes e depois, que permitiu que as mesmas pessoas respondessem aos questionários imediatamente antes e durante a pandemia de covid-19 no Brasil.

Em conclusão, destacamos o predomínio do ganho de peso sobre a perda de peso durante a pandemia da covid-19. Além disso, destacamos o maior risco de ganho ou perda de peso entre pessoas mais jovens, homens e entre aquelas que entraram na pandemia com excesso de peso e o maior risco de ganho de peso entre pessoas com menor escolaridade.

## Footnotes

a World Health Organization. Coronavirus disease (COVID-19) pandemic [Internet]. Geneva: World Health Organization; 2020 [citado 15 dez 2020]. Disponível em: https://www.who.int/emergencies/diseases/novel-coronavirus-2019

b Núcleo de Pesquisas Epidemiológicas em Nutrição e Saúde. NutriNet Brasil. São Paulo: Faculdade de Saúde Pública, Universidade de São Paulo; c2018-2020 [citado 15 dez 2020]. Disponível em: https://nutrinetbrasil.fsp.usp.br
